# User-Friendly and Parallelized Generation of Human Induced Pluripotent Stem Cell-Derived Microtissues in a Centrifugal Heart-on-a-Chip

**DOI:** 10.1089/ten.tea.2019.0002

**Published:** 2019-05-10

**Authors:** Oliver Schneider, Lisa Zeifang, Stefanie Fuchs, Carla Sailer, Peter Loskill

**Affiliations:** ^1^ Fraunhofer Institute for Interfacial Engineering and Biotechnology IGB, Stuttgart, Germany.; ^2^ Faculty of Medicine, Research Institute for Women's Health, Eberhard Karls University Tübingen, Tübingen, Germany.

**Keywords:** organ-on-a-chip, microphysiological system, heart-on-a-chip, engineered cardiac tissue, centrifugal microfluidics

## Abstract

The persistence of cardiovascular diseases as leading global causes of death has spurred attempts to develop microphysiological systems integrating engineered cardiac tissue. These novel platforms enable investigation of mechanisms underlying myocardial pathology as well as *in vitro* screening of candidate drugs for possible cardiotoxicity. However, most of the developed systems rely on manual cell injection protocols, resulting in nonstandardized tissue creation and requiring excessive amounts of cells. To address these issues, we present a novel integrated device enabling the parallelized generation of cardiac microtissues based on human induced pluripotent stem cells as well as rat primary cardiomyocytes in an especially designed multichamber system that provides a precisely controlled physiological environment. The next-generation device utilizes a centrifugally assisted cell loading procedure, which enables robust generation of tissues devoid of air bubbles. It requires solely a minimal amount of cells to create uniaxially aligned cardiac muscle fibers, displaying well-aligned collections of sarcomeres. The viability and functionality of myocardial tissues can be maintained for long time periods, while detailed spatial and temporal beating kinetics can be examined by optical means. As proof of concept, the applicability of the system for drug testing was demonstrated, highlighting the potential of this user-friendly and economical centrifugal heart-on-a-chip for future applications in pharmaceutical industry and mechanistic research.

**Impact Statement:**

With the ultimate goal in tissue engineering of approaching *in vivo* functionality as closely as possible, organ-on-a-chip (OoC) systems provide unprecedented game-changing opportunities by enabling creation of perfused three-dimensional tissues. Most of the recently developed OoC systems, however, require complex handling steps. Hence, a large gap still exists between technology development and collection of valuable biological data in a standardized medium- or high-throughput manner. The system presented here bridges this gap by providing a user-friendly framework for the parallelized creation of multiple physiologically relevant tissues, which could be applicable in every laboratory without additional equipment.

## Introduction

Cardiotoxicity remains a major cause of failure during preclinical as well as clinical drug development and is an important reason for postapproval withdrawal of medicines,^1^ thus contributing to the high cost and inefficiency in the current drug development.^2^ Hence, there is an urgent need for novel, physiologically relevant *in vitro* models of the myocardium. The emergence of human induced pluripotent stem cells (hiPSCs) and hiPSC-derived cardiomyocytes (CMs) has inspired novel and unique approaches in disease modeling and drug testing,^3,4^ raising hopes for a revolution in cardiology research and cardiotoxicity testing.^5^

However, initial approaches focusing on CM monolayers in standard dish cultures^6,7^ or on microstructured surfaces^8,9^ did not fully recapitulate the *in vivo* characteristics of myocardial tissue, particularly its anisotropic, three-dimensional (3D) fiber structure and vascularization. The rise of microfluidic organ-on-a-chip (OoC) systems enabled the culture of tissues with a more *in vivo*-like structure in tailored microenvironments featuring a physiological vasculature-like perfusion,^10–12^ resulting i.a. in microphysiological heart-on-a-chip (HoC) systems.^13–15^

So far, HoC systems have largely fulfilled one of the main promises of OoC technology: they provided physiologically relevant model systems recapitulating key aspects of the *in vivo* structure and functionality of cardiac tissue. Integration of sensing and stimulation capabilities further enabled investigation of mechanical or electrophysiological properties of tissues^15–17^ and the application of external stimuli, such as stretching and/or electrical pacing.^14,18,19^ However, a further main promise of OoC technology, that is, the capability for parallelization and—at least—medium-throughput experimentation, has not been realized.^20^ Although most of the developed HoC systems feature micron-sized footprints, they still consist of single units that require manual cell injection and handling.

Thus, existing systems only permit low-throughput experimentation, requiring expert handling skills for their operation. The postmitotic character of CMs constitutes an additional challenge: to generate 3D tissues with a physiological cell density, high initial cell loading densities have to be achieved, requiring the use of large numbers of cells (typically between 10^5^ and 10^6^) for creation of one individual cardiac tissue.^21,22^

In this study, we describe a novel HoC platform that offers user-friendly parallelized generation of multiple, physiologically relevant, hiPSC-derived, scaffold-free cardiac tissues with microscale dimensions (μ-tissues). The HoC platform allows for injection of cells and generation of μ-tissues by means of centrifugal forces. It was designed to achieve very high loading efficiencies, which in turn minimize the number of required cells. Utilization of standard laboratory centrifuges and simplified preparation steps facilitates generation of a large number of μ-tissues based on easily adoptable routines, without the need for highly specialized operating skills.

We developed a user-friendly open-source framework for the analysis of bright-field video microscopy of the μ-tissues, enabling simple readout. To provide a proof of concept, we performed a parallelized culture and functional validation of cardiac μ-tissues created from hiPSC-CMs and from rat primary CMs, demonstrating the applicability of the system for drug testing.

## Materials and Methods

### Cell culture

#### hiPSC-CM differentiation

The hiPSC line, Coriell GM25256 (RRID: CVCL_Y803, Gladstone Institute for Cardiovascular Disease, San Francisco), used in this study was originally derived from a healthy volunteer with a normal electrocardiogram and no known family history of cardiac disease. After thawing, cells were passaged on growth factor-reduced Matrigel (354277 Corning)-coated T25 flasks at a density of 8,000 cells/cm^2^. Subsequently, cells were cultured in Essential 8 (E8; 05990 STEMCELL Technologies) medium (supplemented with 10 μM ROCK inhibitor Y-27632 [RI; Y0503 Sigma-Aldrich]) for the first 24 h after passaging. hiPSCs were passaged at least three times before initiation of differentiation.

Differentiation was achieved using an optimized protocol for the small-molecule manipulation of Wnt signaling adapted from the study by Lian *et al.*^23^ On day-3, hiPSCs were dissociated with Accutase (A6964 Sigma-Aldrich) and seeded onto Matrigel-coated six-well plates at a density of 25,000 cells/cm^2^ in E8 medium (supplemented with 10 μM RI for the first 24 h).

On day 0, the E8 was replaced by RPMI 1640 medium (RPMI; 1185063 Gibco) containing B27 supplement without insulin (B27-I; A1895601 Gibco) and 12 μM of the Wnt agonist CHIR99021 (CHIR; 4423 Tocris Bioscience). Exactly 24 h after adding CHIR, the medium was changed to RPMI/B27-I w/o CHIR. On day 3, the medium was changed to RPMI/B27-I supplemented with 5 μM Wnt inhibitor IWP-2 (3533 Tocris Bioscience). On day 5, the medium was changed to RPMI/B27-I and 2 days later (on day 7) to RPMI 1640 containing B27 complete supplement (B27C; 17504044 Gibco), which was thereafter used for CM culture, and exchanged every second day.

#### Singularization of hiPSC-CMs

hiPSC-CM sheets were first washed in Dulbecco's phosphate-buffered saline (PBS^+^; 1 mL/well; D8662 Sigma-Aldrich) and then incubated in a dissociation buffer (1 mg/mL collagenase II [LS004174 Worthington] with 40 U/mL DNase I [M0303S BioLabs, Inc.] in Hank's balanced salt solution [H9394 Sigma-Aldrich]) for 1.5 h. The dissociation buffer was aspirated and cells were washed in Dulbecco's phosphate-buffered saline without calcium and magnesium (PBS^−^; D8537 Sigma-Aldrich).

After discarding the PBS^−^, hiPSCs are incubated in 0.25% trypsin/EDTA (15400054 Gibco) for a maximum of 5 min until they detach from the surface. The wells were flushed with RPMI/B27C after which the floating cells were transferred to a 50-mL centrifuge tube (Greiner Bio-One) and centrifuged for 3 min at 138 × *g*. The pellet was then resuspended to the final loading concentration in RPMI/B27C media, enriched with 10 mM RI.

#### Cor.4U cardiomyocytes

Precultured hiPSC-derived CMs were purchased from Ncardia (Cor.4U^**®**^, RRID:CVCL_Y550 Ncardia). They consist of 60% ventricular, 30% atrial, and 10% nodal cells according to the cell provider. Cells were cultured using Cor.4U complete culture medium (Ax-M-HC250 Ncardia) and detached following supplier's protocol.

#### Rat cardiac myocytes

Neonatal, ventricular, Clonetics™ rat cardiac myocytes (R-CM-561) were purchased from Lonza Pharma and Biotech (Basel). Microtiter plates and chips were coated according to manufacturer's instructions with nitrocellulose, and the cardiac myocytes were cultured after thawing for an initial 4 h, using the RCGM BulletKit™ (CC-4515 Lonza) consisting of rat cardiac myocyte basal medium (CC-3275 Lonza) and the RCGM SingleQuots™ Kit (CC-4516 Lonza). After incubation for 4 h, 80% of the medium was removed and replaced with fresh medium that contained 200 μM bromodeoxyuridine (BrdU; CC-4519 Lonza). Culture was prolonged by replacing 50% of the medium supplemented with 200 μM BrdU once every 3 days.

#### Chip fabrication

The centrifugal HoC consists of two microfluidic modules sandwiching a track-etched polyethylene terephthalate (PET) membrane with a pore size of 3 μm (030444 SABEU), which was plasma coated as previously described.^24^ Individual modules were fabricated using standard soft lithography and replica molding techniques.^25^ Briefly, silicon wafer masters for the two modules, featuring microchannels and chambers with heights of 100 μm, were manufactured by UV lithography, using photoresist (SU-8 50 MicroChem) according to the manufacturer's protocols. Subsequently, chlorotrimethylsilane (386529 Sigma-Aldrich) was vapor deposited on wafers for 2 h to facilitate release of polydimethylsiloxane (PDMS).

PDMS (Sylgard 184 Dow Corning) was mixed in a 10:1 (base-to-curing agent) ratio, degassed, and poured onto the wafers. Following overnight curing at 60°C, the PDMS slabs were peeled off the silicon wafer and cropped to chip size. Inlets for the medium channels were punched perpendicularly into the medium layer with a biopsy puncher (504529 World Precision Instruments). The inlet of tissue channels was generated by punching a slanted channel with an angle of 15° (relative to the surface of the upper chip).

Before bonding, PDMS modules were cleaned with isopropanol and by removal of residual particles with Scotch tape. PET membranes were cut using a CO_2_ laser cutter (VLS2.30, Universal Laser Systems) to the desired size and cleaned in ethanol. The two PDMS modules were then treated with O_2_ plasma (15 s, 50 W; Zepto, Diener) and bonded to the PET membrane (structured side of the medium module) and a microscope glass slide (unstructured side of the tissue module), followed by a 2 h bake at 60°C. Media and tissue modules were then bonded together using another O_2_ plasma treatment (15 s, 50 W), thereby sandwiching the membrane. Before use, the chips were sterilized by a 4 min exposure to O_2_ plasma.

#### Loading protocol

To ensure adequate cell attachment, the surfaces of the tissue module were first coated with fibronectin (20 μg/mL in PBS^−^, 33010018 Gibco; both hiPSC-CM types) or nitrocellulose (1-cm^2^ sheet in 10 mL methanol, 10401316 Whatman; rat CMs). A 200 μL pipette tip (Eppendorf), filled with 10 μL of the coating solution, was inserted into the slanted tissue inlet on the edge of the chip, the medium in- and outlets were sealed with stainless steel plugs, and the entire chip was placed in a 50-mL conical centrifuge tube (Eppendorf). The system was then centrifuged at 200 × *g* for 3 min, followed by incubation for 2 h.

For venting, the pipette tip was filled with 100 μL of the appropriate cell culture medium for each cell type, after which the system was centrifuged at 200 × *g* for 3 min. Cell suspensions with a concentration of 10^6^ CMs/mL were prepared according to the singularization protocols described above. One hundred microliters of the cell suspension (corresponding to 12,500 cells per individual tissue chamber) was pipetted into the inserted pipette tip, followed by centrifugation of the chip at 400 × *g* for 10 min.

#### Chip culture

For long-term culture, systems were placed in a CO_2_-controlled incubator (5% CO_2_, 37°C, 95% relative humidity; Binder). The medium channel was continuously perfused with RPMI/B27C (supplemented with RI for 24 h; GM25256), Cor.4U complete culture medium (Cor.4U), or the RCGM BulletKit containing 200 μM BrdU (Rat CMs), at a constant flow rate of 50 μL/h, by an external syringe pump (LA-190 Landgraf HLL), while the medium outflow was collected in a waste receptacle. Tissues were imaged regularly using an inverted light microscope (Leica DMi8) with an integrated incubator.

### Staining

#### Live/dead staining

Live/dead staining solution was prepared by mixing PBS^−^, propidium iodide (PI; 1 mg/mL in PBS^−^; P4170 Sigma-Aldrich), and fluorescein diacetate (FDA; 1 mg/mL in acetone; F7378 Sigma-Aldrich) at a ratio of 30:6:1. Tissues were washed in PBS^−^ by flushing the medium channels and subsequently incubated with the staining solution for up to 15 min depending on cell number. Subsequently, the tissues were washed again with PBS^−^ and then imaged using fluorescence microscopy (Leica DMi8).

#### Immunofluorescence staining

For whole-mount immunofluorescence staining, a previously established protocol was adopted.^26^ Tissues were fixed at room temperature for 15 min, using a 4% solution of Roti^®^-Histofix (P087 Carl Roth), and then permeabilized for 15 min with 0.1% Triton X-100 (28314 Thermo Fisher Scientific). After blocking for 1 h with 3% bovine serum albumin (A9418 Sigma-Aldrich), systems were incubated overnight at 4°C with primary antibodies for sarcomeric α-actinin (ACTN2, clone EA-53, HPA008315 Sigma-Aldrich, dilution 1:1000) and then incubated overnight at 4°C with secondary antibodies (Alexa Fluor 488; A32723 Thermo Fisher Scientific, dilution 1:500). Additionally, nuclei were stained using DAPI (1 mg/mL; D9542 Sigma-Aldrich) diluted 1:500 in PBS^−^ with an incubation time of 30 min.

In between steps, tissues were flushed thoroughly with PBS^−^. To obtain optimal imaging conditions, cardiac chips were taken apart by removing the medium layer. As the membrane still remained on the tissue layer and kept the tissues in place, the chip could be flipped over onto a 170 μm thick cover glass and imaged with a magnification of up to 63 × using a laser scanning microscope (LSM 710; Zeiss).

#### Medium flow and nutrient supply simulation

Medium flow and transport of nutrients were modeled using COMSOL Multiphysics (COMSOL). The membrane was treated as a porous region with a hydraulic permeability of 1.45 × 10^−14^ m^2^ and a porosity of 0.056, similar to previous work.^27^ The flow was described by the Navier–Stokes equation in the free region and the Brinkman equation in the porous region, assuming a water-like medium and a total medium flow rate of 13.9 × 10^−12^ m^3^/s (50 μL/h). To include the diffusive transport of diluted species through the membrane, a time-dependent study using the “Transport of Diluted Species in Porous Media” module was performed, assuming a constant inflow of medium with a concentration of 1 mol/m^3^ and a diffusion coefficient of 1 × 10^−9^ m^2^ for biological molecules in water.

#### Optical analysis of beating rate

Videos of beating cardiac tissues were captured at a rate of 17 fps, and their optical flow was determined by the Python-based open-source tool, *OpenHeartWare*, optimized from a previously introduced approach.^13^

The software possesses a user-friendly GUI and can be downloaded and used for own experimental data analysis.* Each image of size *X* × *Y* in a series of *N* frames was partitioned into a grid of macroblocks with a commonly used size of 16 × 16 pixels. To estimate the motion between frame *i* and a later frame *i* + *s*, for each macroblock in frame *i*, its best matching position within a specific distance *d* from its original position was determined in frame *i* + *s*, leading to a (*N − s*) × *X*/16 × *Y*/16 vector field. As best matching position, we defined the macroblock for which the sum of all squared pixel intensity differences was minimal.

## Results

### Concept of the centrifugal HoC system

**FIG. 1.**
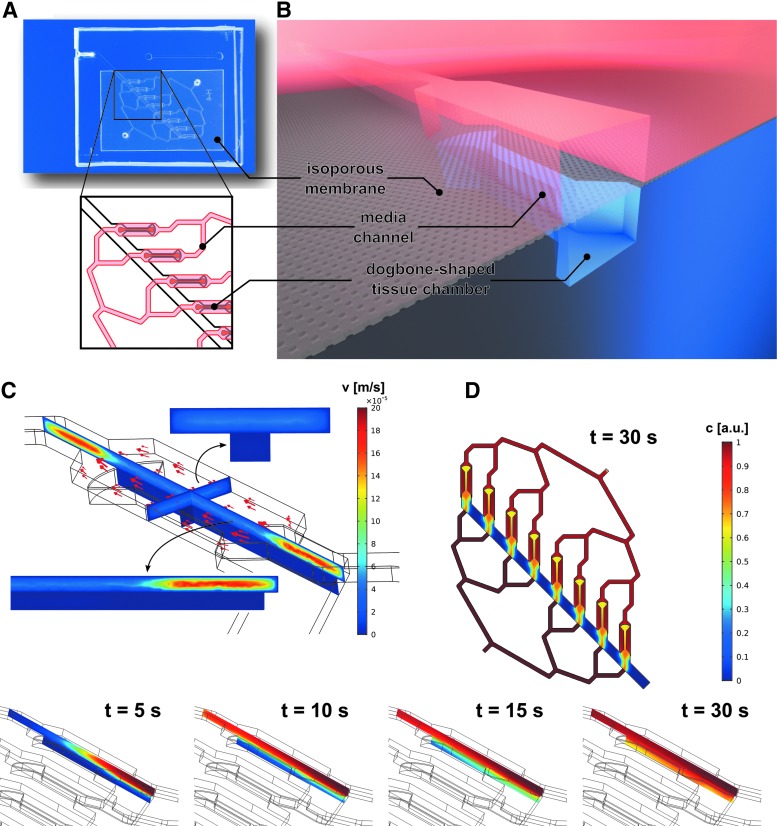
Concept of the centrifugal HoC: **(A)** Photo of the HoC platform featuring a main channel tilted by 45° with respect to the central axis and multiple parallel, dog bone-shaped tissue chambers. The zoomed-in *top view* highlights the parallelized medium supply of individual chambers. **(B)** Detailed *side view* illustrating the employed multilayer approach. hiPSC tissues are cultured in the lower tissue module, separated by an isoporous membrane from the upper medium channels. **(C)** Simulated velocity profile of the medium supply. No additional shear forces act on tissues in the tissue chambers because of the confinement of convective transport to the separate medium module. **(D)** Simulated concentration profile at *t* = 30 s after starting the medium flow. Diffusion of nutrients is equal for all tissues and occurs in the desired subminute temporal range, enabling culture of viable tissues. The exact temporal evolution is shown for one tissue chamber at *t* = 5, 10, 15, and 30 s. hiPSC, human induced pluripotent stem cell; HoC, heart-on-a-chip.

The centrifugal HoC hosts the capability to generate eight individual cardiac tissues. All tissue chambers branch off a main channel (500 μm wide, 100 μm high) that is tilted by 45° with respect to the central axis, that is, the axis parallel to the longer side of the rectangular chip outline, and are arranged parallel to the referred axis (Fig. 1A). The tissue chambers are designed in a characteristic dog bone-shaped geometry with a 150 μm wide and 1 mm long shaft, favoring the formation of an elongated cardiac muscle strand as reported previously^28^: being offered a large adhesion area at both ends, cells can attach at the endpoints and form a connecting fiber in the middle shaft region, thereby acting as a physiologically relevant model system of a myocardial subunit that consists of multiple aligned fibers.^29^

Medium channels are located directly above the tissue chambers and are arranged in such a way that all chambers are perfused with the same amount of medium (Fig. 1A, B). An isoporous membrane acting as an endothelial-like barrier separates the two layers and enables a fluidic connection between medium channels and tissue chambers. Numerical simulations of the velocity profile in defined geometry (Fig. 1C) confirmed the confinement of the convective flow to the medium compartment, thus protecting cultured tissues from additional external forces. Due to porosity of the membrane, cultured tissues are supplied with nutrients by diffusive transport, as verified by the simulated concentration profile (Fig. 1D). The concentration in tissue chambers trailed that of the medium channel with a subminute delay, indicating effective transportation of soluble compounds, such as nutrients or administered drugs.

**FIG. 2.**
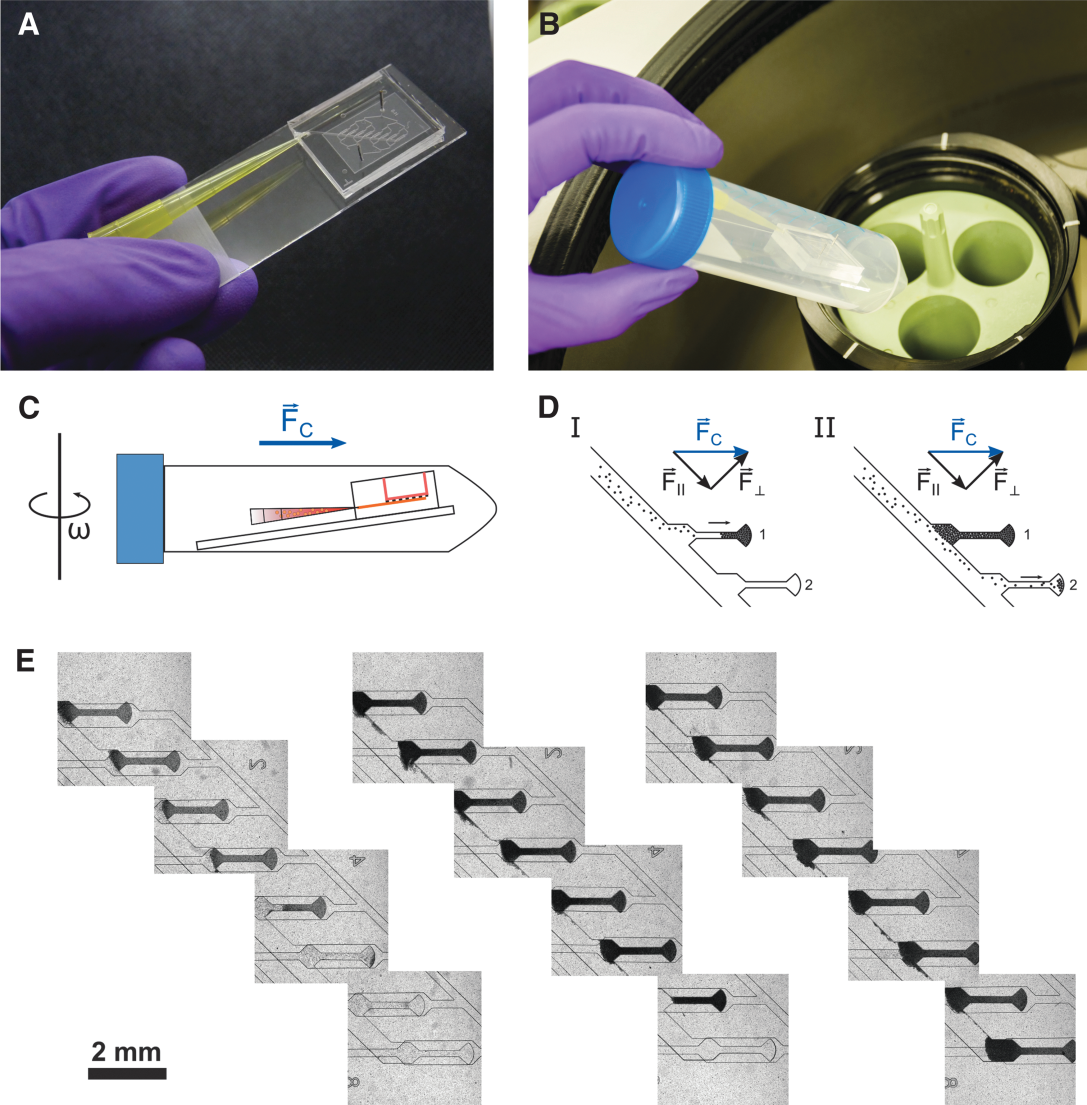
Loading procedure of the centrifugal HoC: **(A)** Photo of an assembled centrifugal HoC system before the loading process. Medium in- and outlets are reversibly sealed by stainless steel plugs, while a pipette tip is utilized to introduce the cell suspension into the chip. **(B)** Photo of chip insertion into a common laboratory centrifuge. A centrifuge tube conveniently acts as sterile interface for accessing the centrifuge. **(C)** Schematic of the system during centrifugal cell injection. During centrifugation with rotational speed ω, the tube is tilted into a horizontal position and the centrifugal force transports cells into the chip. **(D)** Detailed illustration of the filling mechanism. As the main channel is tilted by 45°, individual tissue chambers are filled subsequently in a self-organized metering manner. **(E)** Stitched images of centrifugal HoCs loaded with varying amounts of cells (GM25256, different geometry of the medium module used here). Introducing a sufficient amount of cells leads to eight filled chambers (*right image*), whereas loading with fewer cells leads to fewer generated tissues (*left* and *middle images*); however, all individual tissue chambers are filled entirely, there is no waste of cells.

The centrifugal HoC was mounted on a microscope slide for convenient handling (Fig. 2A). The whole system is designed such that during the loading process the chip can be placed into a conventional centrifuge, transporting the cells into individual tissue chambers by centrifugation instead of conventional external pumps. A pipette tip, which is inserted into the chip at an angle of 15° with respect to the microscope slide plane, allows an easy interface for inserting a cell suspension into the main channel and remains inserted in the chip during the centrifugation step. During centrifugation, medium in- and outlets are reversibly sealed to prevent any convective flow and the entire chip is conveniently placed into a 50 mL conical centrifuge tube that ensures a tight-fitting and, additionally, sterile process environment (Fig. 2B).

One major advantage of cell injection through centrifugal forces is the removal of air bubbles, thereby providing a well-defined system. Before introducing cells, a venting step is carried out during which the pipette tip is only filled with media and subsequent rotation ejects remaining air bubbles from the chip. While the pipette tip is still mostly filled with media, subsequent injection of the cell suspension does not introduce new air bubbles, ensuring a robust cell loading process during centrifugation (Fig. 2C).

The basic property of centrifugal force, that is, its directionality in contrast to isotropic forces during conventional pressurized loading, allows for its exploitation for novel loading concepts. As cells are introduced into the tissue module, the tilted channel design ensures that cells (which are transported radially outward in the completely air-free channel) accumulate, due to their density, at the distal channel wall during centrifugation. The centrifugal force acting on cells may be decomposed into one component parallel to the channel wall and one component perpendicular to it (Fig. 2D). As long as the wall compensates the perpendicular force component, cells are driven radially outward along the radially outer-lying channel edge.

As cells reach the first tissue chamber, they are trapped and start filling the 3D chamber completely, from the radially distal-lying parts upward, that is, toward the chamber entrance. Only after the first chamber has been filled entirely, consecutive cells will travel further downstream and start filling the next chamber. This ensures that during the loading procedure, cells fill consecutive chambers in a self-organized metered behavior, leading to uniformly filled, densely packed tissue chambers. Centrifugation parameters were optimized to the lowest centrifugal force still able to reliably and repeatedly fill the tissue chambers.

If the proper amount of cells is loaded, all filled tissue chambers contain the same amount of cells, whereas the intermediate channel is free of cells, leading to generation of independent, nonconnected, cardiac muscle strands during further culture. On reducing the number of introduced cells (Fig. 2E), one of the key advantages of the used geometry becomes evident: even if the volume of the introduced cell suspension is insufficient to fill all chambers, the chip and especially the deployed cells are not wasted because the described approach leads to consecutive loading of individual chambers, still creating multiple identical—although fewer—tissues.

Other approaches in which the total amount of cells is evenly distributed between all chambers would lead to chambers only partially filled with cells, precluding its use for further comparable and standardized tissue analysis. If excessive cell numbers are injected and the volume of introduced cells exceeds the added up volume of all tissue chambers, the main channel fills up, leading to a short circuit by connecting neighboring tissues (Supplementary Video S1).

Utilizing a slightly different chip design with modified loading channel geometry, the centrifugation-based loading concept also opens up the possibility of generating defined cocultures by consecutive loading steps (Supplementary Fig. S1).

### Tissue formation and culture

**FIG. 3.**
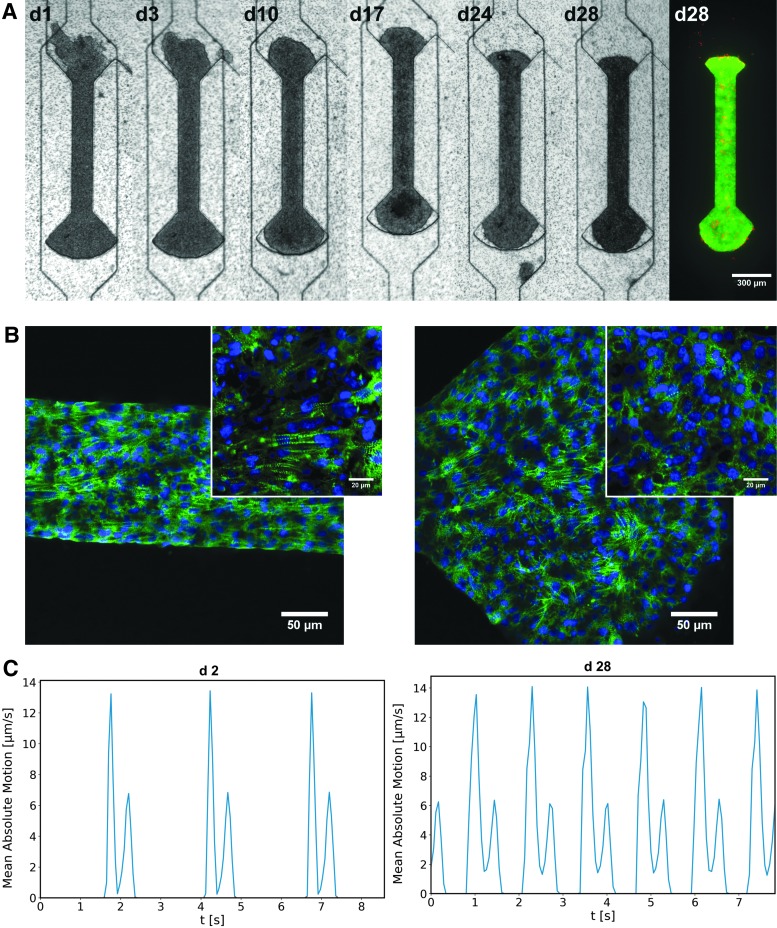
Formation of cardiac μ-tissues (GM25256): **(A)** Development of hiPSC-derived cardiac μ-tissue over the time span of multiple weeks. After cell seeding, the tissue compacts and starts beating uniaxially, still viable after 4 weeks, as indicated by live/dead staining (*green*: FDA, *red*: PI). **(B)** Physiological validation of cultured hiPSC-derived cardiac μ-tissues by immunostaining of α-actinin (*green*) and DAPI (*blue*), fixed on day 28 after seeding (25× magnification). The *insets* show a zommed in view of the central region (63× magnification). Horizontally aligned sarcomeres prevail in the shaft region (*left*), whereas sarcomeres are randomly oriented in the knob region (*right*). **(C)** Beating kinetics of the investigated tissue on days 2 and 28, revealing an increase in beating rate. FDA, fluorescein diacetate.

After cell seeding, medium plugs as well as the pipette tip used for cell insertion were removed, and the tissue inlet was blocked. A syringe pump was connected to the medium inlet, a waste container to the outlet, and the tissues were supplied with media at a flow rate of 50 μL/h. Generally, the cultured cardiac tissues began beating spontaneously on day 2 after seeding (Fig. 3C; Supplementary Video S2). During on-chip tissue formation, single CMs connect to an aligned tissue fiber and synchronize their beating pattern (Fig. 3A). Cardiac μ-tissues from hiPSC-CMs could be cultured over multiple weeks and were still showing a pronounced beating motion 4 weeks after initial seeding (Fig. 3C; Supplementary Video S3).

To assess tissue viability, live/dead staining was performed, confirming that even after 4 weeks of culture, the bigger part of the tissue was viable and dead cells occurred only sporadically, indicating an intact tissue (Fig. 3A). For further physiological validation, immunofluorescence staining of sarcomeric α-actinin was conducted and analyzed by confocal microscopy (Fig. 3B). The microscopy images reveal characteristically striped cytoskeletal structures (α-actinin) as well as a parallel alignment of sarcomeres, particularly in the shaft region of the tissue chambers. The sarcomeres within the shaft region show a preferred alignment parallel to the main axis of the tissue, in contrast to the knob region where sarcomeres of the substrate-adhered tissue were rather unorganized and did not show a distinct orientation.

**FIG. 4.**
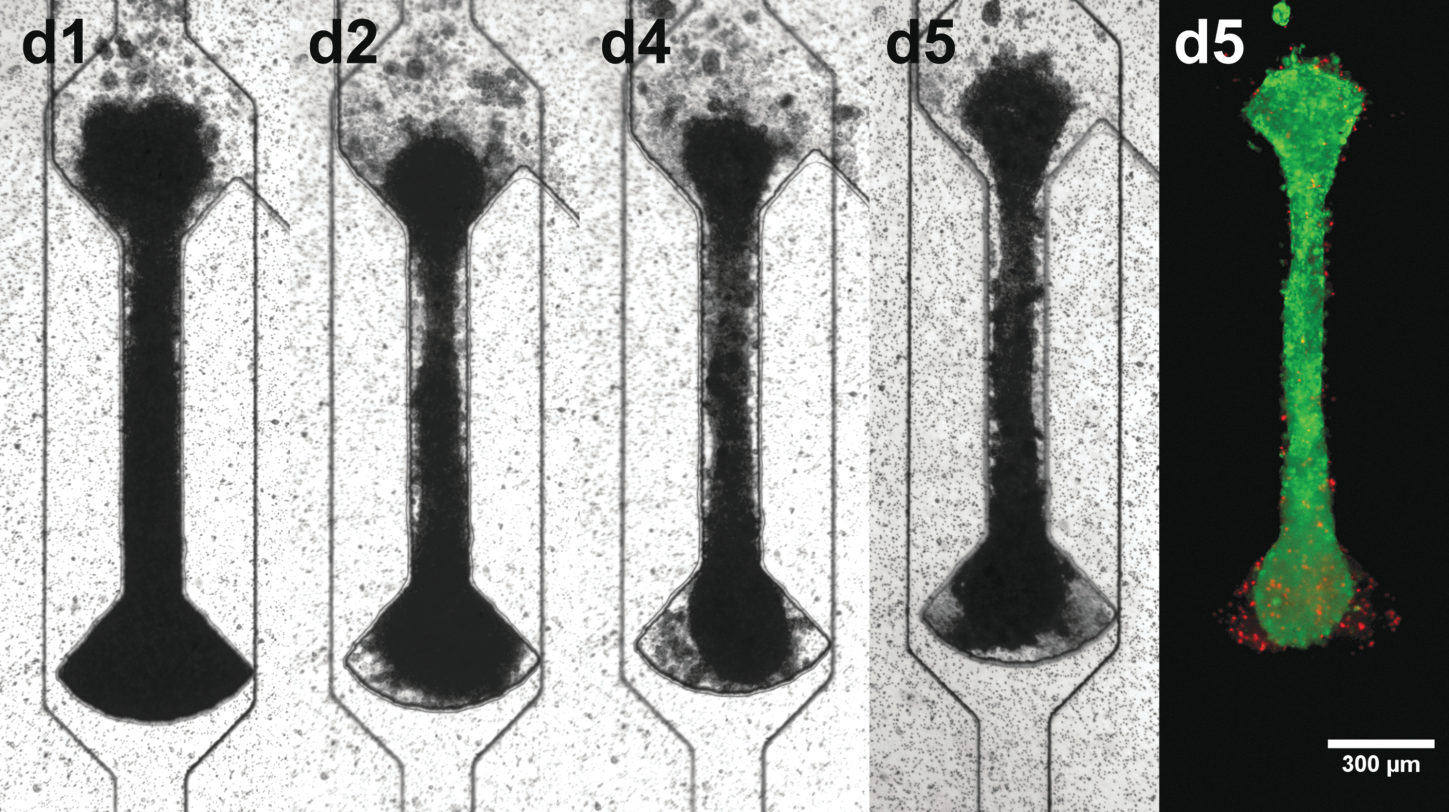
Universal applicability of developed centrifugal HoC: development of μ-tissue from rat CMs. Tissues show the same fiber formation as with hiPSC-CMs and are viable and functional in the centrifugal HoC, indicated by physiological beating and live/dead staining (*green*: FDA, *red*: PI). CM, cardiomyocyte.

To show the versatility of the presented system, the centrifugal HoC was utilized to generate and culture cardiac μ-tissues from rat primary CMs. Similar to hiPSC-CMs, rat CMs formed viable and functional fiber structures (Fig. 4). However, the temporal as well as spatial dynamics were different; in contrast to hiPSC-based tissues, rat tissues showed a nonuniform, random beating pattern (Supplementary Video S4).

### Optical flow-based analysis

**FIG. 5.**
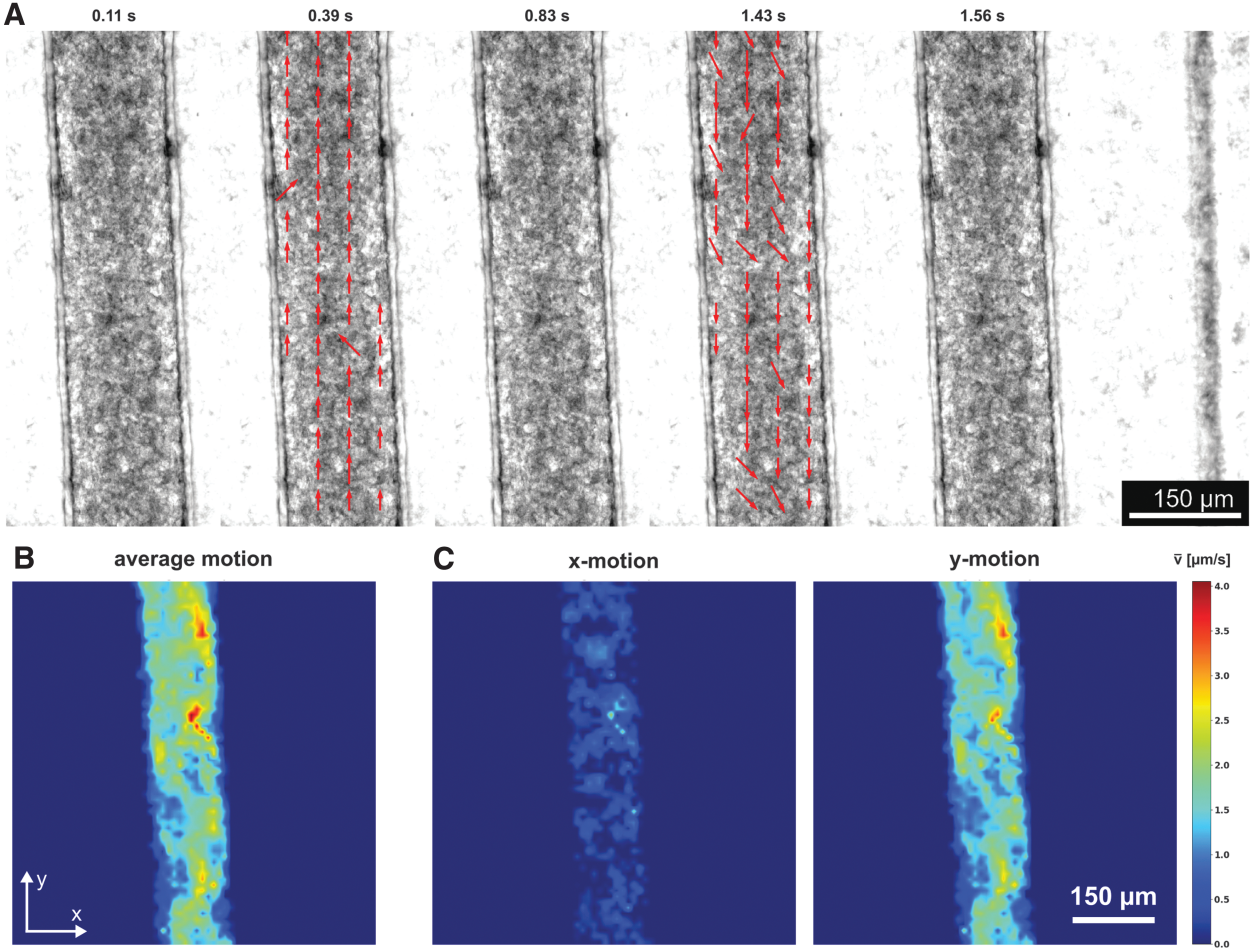
Analysis of beating pattern based on video microscopy (GM25256, d6): **(A)** Quiver plots at various time points illustrating the space-resolved tissue movement indicated by *red arrows*. At *t* = 0.39 s, a predominantly parallel downward motion is observable, whereas at *t* = 1.43 s, the tissue contracts again in the opposing direction. **(B)** Heat map expressing the time-averaged absolute tissue movement, revealing tissue movement in the complete shaft. **(C)** Heat maps of the decomposed absolute tissue movement for *x*- and *y*-movements indicating a predominant direction of contraction along the channel axis (*y*-axis).

Functional tissues could be optically distinguished by their noticeable beating motion. To quantify beating kinetics, video microscopy was used and the recorded movies analyzed using our newly developed open-source software, *OpenHeartWare*. This specifically tailored software is based on optical flow motion tracking algorithms similar to previous approaches.^13,30,31^ The analysis yields a time-dependent displacement vector field, which serves to characterize and compare beating patterns of individual tissues. The obtained time-dependent vector field may be represented as a quiver plot that can be used as an overlay to the raw video recording to visualize the beating behavior more clearly (Fig. 5A; Supplementary Videos S1–S7).

Thus, during all phases of periodic contraction and relaxation, the coordinated movements in tissue regions could be monitored accurately against the nonmoving background. The beating velocity was further spatially averaged and shown as a function of time (Fig. 3C), resulting in a periodic beating pattern in which contraction peaks and beating rate could be detected with ease. An exemplary analysis of the beating behavior of the hiPSC-based μ-tissues (GM25256) at day 2 (24 ± 0.3 bpm) and day 28 (47.1 ± 0.8 bpm) shows an increase of the beating rate to a physiological range (Fig. 3C; Supplementary Videos S2 and S3).

For further analysis of the spatial distribution of beating in tissues, the displacement velocity was averaged temporally over the complete recorded time frame (Fig. 5B) and decomposed into its *x* and *y* movement components (Fig. 5C). A spatial distribution of average displacement velocities ranging from 1 to 4 μm/s could be determined in the tissue. Comparisons of *x* and *y* movements revealed a clearly dominating *y* movement in the shaft region, confirming creation of a physiologically relevant, uniaxially beating tissue strand.

### Functional characterization

**FIG. 6.**
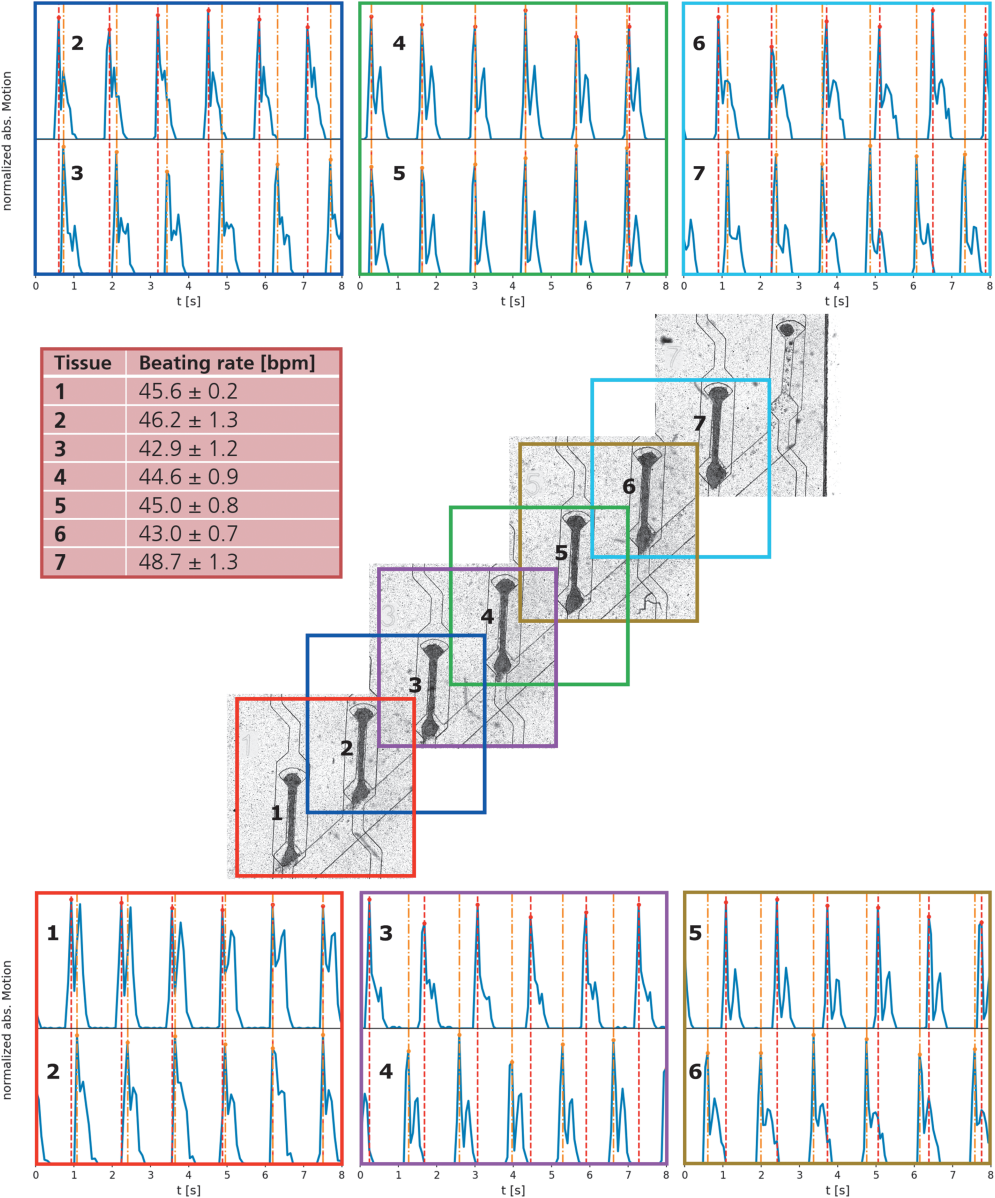
Correlation between generated μ-tissues: stitched image of a centrifugal HoC on day 5, hosting seven hiPSC-derived cardiac μ-tissues (Cor.4U^®^). In each colored region, the beating behavior of two neighboring tissues is analyzed simultaneously using video microscopy and depicted pairwise for comparison (*top* and *bottom*). In each panel, peak positions are marked for comparison (upper peaks: *red*, lower peaks: *orange*). All tissues display an independent beating behavior decoupled from their neighbor as their contraction peaks do not overlap. Individual beating rates lie in a close physiological range of 45 ± 2 bpm (*left center*).

To verify the independence of individual μ-tissues within one chip, pairwise videos were recorded of neighboring tissues on a chip hosting 7 μ-tissues (Cor.4U) on day 5 (Fig. 6; Supplementary Video S8). Thereby, individual beating kinetics could be related to each other. All tissues beat within the same physiological range of 45 ± 2 bpm. Neighboring tissues are, however, not synchronized in their beating behavior as contractile peaks occur at different time points.

**FIG. 7.**
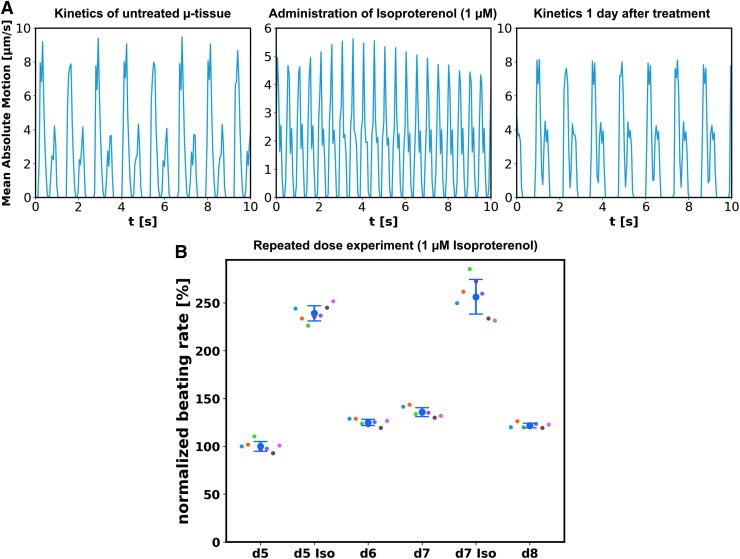
Drug testing in hiPSC-derived cardiac μ-tissues: **(A)** Beating kinetics extracted by optical means of a μ-tissue (GM25256) before and during administration of 1 μM isoproterenol (recorded 5 min after inclusion of isoproterenol into the medium, both day 6), as well as 1 day after drug administration. An increased beating rate can be clearly recognized after drug administration, indicating the suitability of the system for compound testing. **(B)** Optically extracted beating rate from seven tissues (Cor.4U^®^) within one chip, normalized to the mean beating rate on day 5. Individual tissues as well as the mean values are depicted. Isoproterenol (1 μM) was administered on days 5 and 7 through the culture media. Isoproterenol treatment results each time in a pronounced beating rate increase with a decline to physiological beating rates on the following day.

Functional characterization of the system was performed by treatment with the selective β-adrenergic agonist, isoproterenol. The perfusion medium was supplemented with isoproterenol (1 μM) and delivered through the medium channel. Beating kinetics extracted by means of optical flow of a representative μ-tissue (GM25256) after 6 days of culture, before, during, and 1 day after isoproterenol treatment (Fig. 7A; Supplementary Videos S5–S7), revealed an initial increase in the beating rate and subsequent recovery of baseline beating after washout: the beating rate increased within 5 min from 46 ± 1 to 120 ± 3 bpm, representing an increase by 160% ± 12%, and relaxed on the following day to the initial beating rate again.

In a repeated dose experiment, isoproterenol (1 μM) was administered on days 5 and 7 after loading to a chip hosting seven functional μ-tissues (Cor.4U). The beating rate was measured for each individual tissue before and 5 min after drug administration, as well as after washout of the drug in the unperturbed state on days 6 and 8 (Fig. 7B). Isoproterenol administration on day 5 led to an increase in relative beating rate to 239% ± 8%, which relaxed to 125% ± 3% (day 6) and 136% ± 5% (day 7). Subsequent drug administration led to an increase to 256% ± 18%, which relaxed to 122% ± 2% (day 8).

## Discussion

Engineered cardiac tissues based on hiPSC-derived CMs have been extensively studied over the past decade, thereby various experimental models evolved providing tools for evaluating and manipulating functional, electrophysiological, or mechanical performance, each system tailored to addressing a specific question. The aim of the present study was to develop a microphysiological HoC platform for convenient, robust, and efficient generation and characterization of cardiac μ-tissues in a parallelized manner.

The novel centrifugal loading procedure described here represents a general user-friendly approach, which is broadly applicable, and does not require additional manual handling steps or external components (e.g., bubble traps) other than a conventional centrifuge that may be found in any cell culture laboratory. The method is based on physical principles ensuring a robust, that is, reproducible, performance facilitating automation of procedural steps. The application of centrifugal forces during cell loading exploits their unidirectionality, which aids the crucial removal of trapped air bubbles due to their low density, as well as leads to consecutive loading of multiple chambers in a self-organized manner.^32,33^

As mentioned above, an essential characteristic of our system is the high loading efficiency: this ensures the delivery of all introduced cells into separate chambers and formation of equal-sized individual tissues, which is crucial when working with cost-intensive patient-specific hiPSC-CMs on a large scale, for example, in future industrial applications. As proof of concept, a system containing eight cardiac μ-tissues was presented; however, the individual chip design could be further extended to include an arbitrary number of tissue chambers as well as easily tailored to other cell types.

Due to the sub-mm^2^-dimensioned tissue footprints, a well plate-sized system hosting hundreds of μ-tissues would be technically feasible. Furthermore, the designed multilayered structure of the microphysiological platform permits facile integration of additional readouts or stimuli for investigating the generated tissues.

As the human heart consists of bundled, uniaxially contracting muscle fibers, an authentic physiological model should embody a similar structure. By tailoring the chamber design to a dog bone-like shape for cardiac μ-tissues, mentioned contraction orientation is induced. The presented centrifugal HoC provides vasculature-like perfusion while protecting tissues from shear forces by an isoporous membrane, as shown by both simulations and experiments.

Cell loading through centrifugation with an acceleration of 400 × *g* for 10 min did not induce evident adverse effects on loaded cells and led to viable and functional beating tissues. The developed concept further paves the way for unprecedented approaches to, for example, tune cellular density with rotation speed or to perform a precise coloading with different cell types. Should the amount of introduced cells be too low, the inserted pipette tip offers an easily accessible interface to conduct successive loading steps.

Although the envisioned application and future development of the platform will probably be focused on hiPSCs because of their immense potential for disease modeling and personalized medicine, we confirmed the compatibility of the system with other cell types: we successfully generated cardiac μ-tissues based on primary rat CMs by centrifugal loading and cultured them in the HoC. Cultured μ-tissues were not only viable as verified by live/dead staining but also possessed a physiologically relevant, elongated fiber shape, as indicated by aligned sarcomeres in the shaft region of the tissue chambers.

In addition, tissues that were cultured in the system for a time span of more than 1 month still showed a viable beating motion. This opens up the possibility for future studies aiming at creating advanced, matured, hiPSC-derived cardiac μ-tissues on chip, constituting a physiologically more relevant model system.

Moreover, a further aspect that could be the focus of future development is the refinement of the endothelial-like barrier between perfusing media and tissue chambers. The currently employed artificial porous membrane succeeds in protecting tissues from nonphysiological shear forces while at the same time providing a sufficient nutrient supply through diffusion. However, it does not recapitulate biological transport processes of the endothelial barrier *in vivo*. This important aspect may be added by lining the medium channel-facing side of the membrane with endothelial cells (proof of concept cf. Supplementary Fig. S2).

For noninvasive investigation of beating kinetics of individual tissues, we provide the easy-to-use open-source tool, *OpenHeartWare*, which allows determination of spatial and temporal dynamics of arbitrary cardiac tissues solely based on bright-field video microscopy. Applying this tool to hiPSC-derived cardiac μ-tissues, the desired mimicry of a myocardial fiber with uniaxial contractile motion could be validated in the shaft region. Measured beating velocities are of the order of magnitude of 10 μm/s comparable with other similar systems.^14,26,32^

As proof of concept for application in drug testing, an increase in beating frequency as a response to administration of isoproterenol was observed, similar to previous studies.^34^ Due to the low number of 12,500 required cells per individual μ-tissue unit, the system could be easily scaled up to perform drug testing even on a much larger scale, with more doses and/or drugs, exploiting the benefit of eight replicates within one chip.

In conclusion, a physiologically relevant HoC system was developed and characterized. It enabled robust loading, culturing, and optical analysis of eight individual, morphologically and functionally complex, miniaturized cardiac μ-tissues, each consisting of only ∼12,500 cells. The developed system thus fulfills all requirements to serve as a platform for various biomedical applications, such as drug testing or personalized medicine.

## Supplementary Material

Supplemental data

Supplemental data

Supplemental data

Supplemental data

Supplemental data

Supplemental data

Supplemental data

Supplemental data

Supplemental data

## Notes

^*^ https://github.com/loslab/ohw
