# Supplementary material for: User-Friendly and Parallelized Generation of Human Induced Pluripotent Stem Cell-Derived Microtissues in a Centrifugal Heart-on-a-Chip
Source: Tissue Eng Part A. 2019 May 10;25(9-10):786–98. doi: 10.1089/ten.tea.2019.0002 (PMC6535963; doi:10.1089/ten.tea.2019.0002)
Supplement: Supplemental data [file Supp_Data.pdf]

Supplementary Data

Supplementary Materials and Methods

Coloading

Cor.4U® hiPSC-derived cardiomyocytes were detached according to manufacturer's instructions and split into two equal parts consisting of 140,000 cells. One part was labeled with CellTracker™ Green CMFDA (C7205, Invitrogen) by resuspending the cells in 1 mL of CellTracker solution (25 μM in RPMI) and subsequent incubation for 45 min. Afterward, the cells were washed in RPMI once and resuspended to the final loading concentration of 160,000 cells/mL in Cor.4U complete culture medium. The other unstained part was directly resuspended to the same concentration in Cor.4U complete culture medium.

After venting a centrifugal heart-on-a-chip (HoC) with modified loading geometry (Supplementary Fig. S1A) according to the loading protocol, 50 μL of the labeled suspension (corresponding to 8,000 cells with a volume to fill two cardiac chambers halfway) was pipetted into the pipette tip, followed by subsequent centrifugation at $400 \times g$ for 10 min. As the actual cell concentration deviated from the calculated concentration to achieve desired filling, this loading step was repeated with 100 μL of labeled cell suspension. In a subsequent loading step, 150 μL of remaining medium was aspirated from the loading pipette tip and refilled with 150 μL of the unlabeled cell population, followed by centrifugation at $400 \times g$ for 10 min. Loaded chips were imaged using fluorescence microscopy (Leica DMi8).

Isolation and culture of primary human microvascular endothelial cells

Dermal microvascular endothelial cells (mvECs) were isolated from an adult human skin biopsy obtained from plastic surgery performed on a female preobese donor (BMI 28.2 as per the WHO classification), aged 49 years, by Dr. Ulrich E. Ziegler (Klinik Charlottenhaus, Stuttgart, Germany). All procedures were carried out in accordance with the rules for medical research of human subjects as defined in the Declaration of Helsinki. Patients signed a written consent form according to the Landesärztekammer Baden-Württemberg (IRB# F-2012-078).

Primary human mvECs were isolated as previously described.[S1] The positive selection of endothelial cells was performed by incubation with 0.02% EDTA solution (17-711E Lonza) to detach possibly entrained fibroblasts. Cells were expanded at a cell density of 28,000 cells/cm^2 in endothelial cell growth medium (C-22010 Promocell) in cell culture flasks and used at passage 2 for seeding into the centrifugal HoC.

mvEC seeding

mvECs were seeded into a centrifugal HoC with viable cardiac μ-tissues (Cor.4U) on day 6 after loading. The medium supply was disconnected from the chip and a 200-μL pipette tip (Eppendorf) inserted into the outlet. Seventy microliters of an mvEC suspension (6×10^6 mvECs/mL) was aspirated into a 200-μL pipette tip, which was subsequently introduced into the medium inlet. By carefully discharging about 10 μL of the pipette tip, the medium channel was flushed with the cell suspension. While remaining inserted into the medium inlet, the pipette tip was disconnected from the pipette and the whole chip incubated for 3 h with a remaining hydrostatic flow. Afterward, the chip was reconnected to the medium supply and cultured using Cor.4U complete culture medium at a flow rate of 50 μL/h.

Supplementary Reference

S1. Volz, A.-C., Huber, B., Schwandt, A.M., and Kluger, P.J. EGF and hydrocortisone as critical factors for the co-culture of adipogenic differentiated ASCs and endothelial cells. Differentiation **95,** 21, 2017.

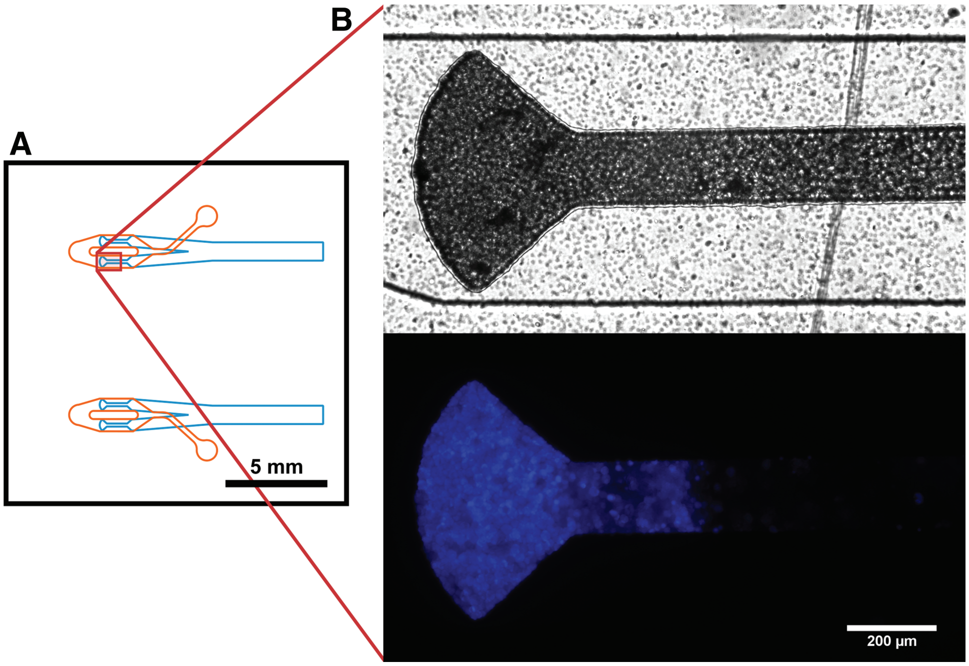

SUPPLEMENTARY FIG. S1. Centrifugal coloading. **(A)** Modified centrifugal HoC geometry featuring an even branching of the main channel (*blue*), optimized for successive loading passes. One chip holds two separate systems, which are perfused by overlying medium channels (*orange*). **(B)** Bright-field image of a cardiac tissue chamber filled with CMs (Cor.4U®, d0, *upper panel*). The loading process was decomposed to first introduce a cell population labeled with a cell tracker dye, followed by the pristine population. Fluorescence microscopy reveals a defined cell distribution (*lower panel*), illustrating the opportunity of the centrifugal HoC to generate precisely controlled cocultivations. CM, cardiomyocyte; HoC, heart-on-a-chip.

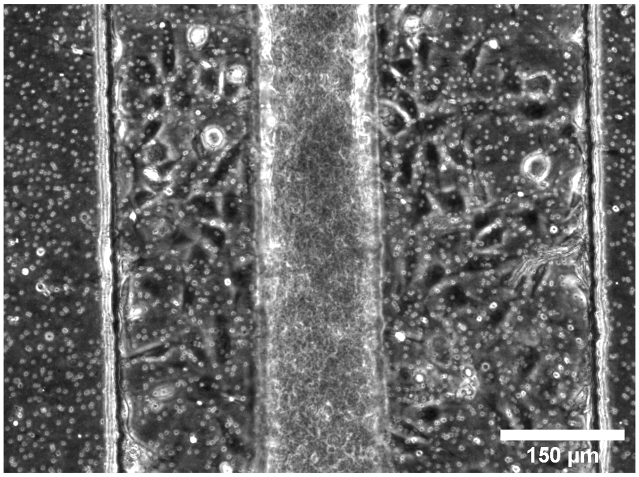

SUPPLEMENTARY FIG. S2. Endothelialization of cardiac µ-tissues: Phase-contrast image of a centrifugal HoC hosting mvECs in the medium channel. The 3D tissue strand (Cor.4U®, d14, center region) is cocultured with an overlying mvEC monolayer (seeded on d6, broad outer channel). 3D, three-dimensional; mvEC, microvascular endothelial cell.
